# Are we systematically overdosing women? Revisiting standardized contrast protocols for thoracoabdominal CT scans

**DOI:** 10.1007/s00330-024-11329-8

**Published:** 2025-01-09

**Authors:** Judith Becker, Adrian Huber, Stefanie Bette, Anna Rubeck, Tim Tobias Arndt, Gernot Müller, Franka Risch, Luca Canalini, Claudia Wollny, Florian Schwarz, Christian Scheurig-Muenkler, Thomas Kroencke, Josua A. Decker

**Affiliations:** 1 Department of Diagnostic and Interventional Radiology, University Hospital Augsburg, Augsburg, Germany; 2 Department of Computational Statistics and Data Analysis, Institute of Mathematics, University of Augsburg, Augsburg, Germany; 3 Clinic for Diagnostic and Interventional Radiology, Donau-Isar-Klinikum, Deggendorf, Germany; 4 Centre for Advanced Analytics and Predictive Sciences, University of Augsburg, Augsburg, Germany

**Keywords:** Photon-counting detector CT, Contrast media, Image enhancement, Sex differences

## Abstract

**Objectives:**

The purpose of this study was to evaluate whether the iodine contrast in blood and solid organs differs between men and women and to evaluate the effect of BMI, height, weight, and blood volume (BV) on sex-specific contrast in staging CT.

**Materials and methods:**

Patients receiving a venous-phase thoracoabdominal Photon-Counting Detector CT (PCD-CT) scan with 100- or 120-mL CM between 08/2021 and 01/2022 were retrospectively included in this single-center study. Image analysis was performed by measuring iodine contrast in the liver, portal vein, spleen, left atrium, left ventricle, pulmonary trunk, ascending and descending aorta on spectral PCD-CT datasets. Univariable and multivariable analyses were performed to assess the impact of sex, age, BMI, height, weight, and BV on the iodine contrast.

**Results:**

A total of 274 patients were included (mean age 68 years ± 12 SD, 168 men). Iodine contrast in organs and blood attenuation was significantly higher in women when using the same volume of CM. Sex, age, BMI, height, weight, and BV significantly influenced iodine contrast. After adjusting for confounding variables, sex remained a significant factor, with women having higher parenchymal and vascular iodine contrast.

**Conclusion:**

Standardized or weight-adapted use of CM in venous-phase thoracoabdominal CT scans results in significantly higher contrast in women compared to men. Customizing the CM dose to the patient’s BV could result in a similar contrast between sexes. This approach has the potential to reduce the amount of CM, resulting in cost savings, and to decrease the risks associated with CM, particularly for the female sex.

**Key Points:**

***Question*** *This study addresses whether current standardized iodinated contrast media protocols lead to systematically higher iodine enhancement in women than in men during thoracoabdominal CT.*

***Findings*** *Women consistently show greater iodine enhancement in blood and abdominal organs compared to BMI-matched men when receiving identical volumes of contrast media.*

***Clinical relevance*** *Adjusting contrast media dosage based on blood volume in venous-phase CT scans could equalize parenchymal and intravascular iodine enhancement across sexes. This approach may reduce unnecessary contrast exposure in women, lower associated risks, and optimize healthcare resource allocation.*

## Introduction

The use of intravenously administered contrast media (CM) in diagnostic imaging has become indispensable for visualizing and evaluating pathologies [1]. The contrast enhancement of these structures depends on many factors, such as the applied volume of CM, injection rate, scan delay, and scan duration [1, 2]. However, the volume of the intravenous CM injected remains a controversial issue. In most cases, the volume applied for optimal contrast is based on body weight or a fixed study-specific dose [2–5]. On the other hand, there are studies suggesting that other patient characteristics, such as lean body weight, body surface area (BSA), or blood volume (BV), can be used to better determine an individualized and minimal amount of CM to achieve optimal iodine contrast on imaging [6–8].

Despite the great benefits of contrast-enhanced imaging, the use of iodinated CM use must be carefully considered because of various factors. Some of the most important risks include allergic reactions, contrast-induced nephropathy and inducible thyroid dysfunction [9–11]. Historically, these risks were thought to be sex-unspecific affecting men and women equally; however, recent studies suggest that women are more likely to experience adverse effects from CM than men [12–14]. Since the probability of adverse reactions is additionally increased with the volume of applied CM, women are potentially exposed to an even higher risk [15–17]. A dose-saving and, above all, dose-conscious use of iodine-containing CM is not only essential with regard to the shortage of resources but can also lead to a substantial reduction in costs in healthcare systems due to their extensive use worldwide [18, 19].

PCD-CT systems enable the routine reconstruction of virtual non-contrast (VNC) images and especially iodine maps using their inherent spectral data [20]. These allow the determination of the proportion of iodine contrast in each CT scan [21]. While conducting a study on anemia detection in PCD-CT, a sex difference in iodine contrast was observed [22]. To our knowledge, no other studies have previously examined sex differences in contrast enhancement with the same amount of CM.

The purpose of this study was to determine whether patient sex significantly influences different enhancements of iodinated contrast using identical administration protocols. In addition, we aimed to identify parameters that could be used for an individualized and sex-specific amount of CM that would result in sex-invariant contrast enhancement.

## Materials and methods

### Patients

This retrospective, observational single-center study was approved by the local Medical Research and Ethics Committee (MREC). Written informed consent was obtained from all study participants (study protocol number 21-0280). Patients with a clinically indicated thoracoabdominal CT scan in portal-venous phase between 08/2021 and 01/2022 on a dual-source PCD-CT system (NAEOTOM alpha, Siemens Healthineers) were included. Patient characteristics and laboratory parameters were obtained from electronic medical records.

Inclusion criteria: (1) clinically indicated thoracoabdominal CT scan with suspected/known malignancy, (2) intravenous application of either 100- or 120-mL CM, (3) acquisition in portal-venous phase, (4) patient age ≥ 18 years, (5) availability of complete patient characteristics (age, sex, height, weight), (6) written informed consent.

Exclusion criteria: (1) different injected CM volume (e.g., due to impaired renal function or very low BMI), (2) status post splenectomy and hemihepatectomy (as no valid measurements can be performed), (3) extensive liver metastases.

### CT protocol

Patients were scanned craniocaudally in supine position from the thoracic inlet to the symphysis during a single breath-hold. The following parameters were applied: automatic tube current modulation (Care DOSE 4D, Siemens Healthineers) with an image quality level of 145 and iterative image reconstruction, spectral acquisition mode (QuantumPlus, Siemens Healthineers), tube voltage of 120 kVp with automatic tube current modulation on (Care DOSE 4D, Siemens Healthineers optimized for soft tissue with contrast), rotation time of 0.25 s, pitch of 0.8, collimation of 144 × 0.4 mm. Spectral series of the whole volume were generated using a soft tissue, specifically kernel (Qr40, QIR 3, Siemens Healthineers) and an enhanced DICOM file format with full spectral information (SPP, Spectral Postprocessing, Siemens Healthineers). The slice thickness was 3 mm with an increment of 1 mm.

The scan protocol used a biphasic contrast injection with 100- or 120-mL iodinated CM (iopromide: Ultravist 300 mgI/mL, Bayer) injected via an antecubital vein followed by a saline bolus of 30 mL (flow rate 4.0 mL/s). The scan started bolus-triggered with a delay of 45 s after reaching an enhancement of 120 HU within the ascending aorta.

Volumetric computed dose index (CTDI_vol_), dose length product (DLP) and size-specific dose estimate (SSDE) were extracted from the automatically generated structured dose reports.

In our department, we mainly use a standardized volume of CM. However, this is reduced in patients with very low BMI or patients with reduced renal function, and they have been excluded from this study. With the new PCD-CT and improved photon detection, we reduced the amount of CM to 100 mL. Other studies also used 100 or 120 mL CM as a fixed dose for an abdominal CT and confirmed that 100 mL of CM is practicable [23–27]. During routine patient workup, all scans were initially reviewed by the attending board-certified radiologists according to our institutional standards (each with at least 5 years of radiology experience) to ensure sufficient contrast attenuation.

### Image analysis

Two senior radiologists with 6 and 7 years of experience analyzed the CT images on a dedicated workstation Syngo.via (VB60A, “MM reading” workflow, Siemens Healthineers) based on spectral post-processing (SPP) series for 70 keV virtual monoenergetic imaging (VMI). Circular regions of interest (ROI) were placed in the following anatomic structures: Liver (right and left lobe), portal vein, spleen, left atrium, left ventricle, pulmonary trunk, ascending and descending aorta (left atrium, left ventricle, pulmonary trunk, ascending, and descending aorta are summarized via the mean value in the following chapters as blood attenuation). ROIs were placed carefully, avoiding larger vessels and focal lesions within organs as well as intracavitary structures or vessel walls within the heart and the aorta. For each ROI, three attenuation values (in Hounsfield units; HU) were obtained in (1) contrast-enhanced images, (2) virtual non-contrast image (VNC), and (3) the pure iodine contrast in iodine maps and respectively used and compared in further analyses. Due to the inherent spectral information of SPP series from PCD-CT datasets, VNC and iodine contrast values are readily available for each series without additional post-processing.

### Statistical analysis

Parameters were descriptively analyzed by means and standard deviations. Homogeneity of variances was evaluated with the Levene test. Differences in means were then further evaluated with the Welch or *t*-test. The contrast values were examined for normal distribution. The association between contrast values and the assessed patient characteristics was analyzed using linear regression. The regression models have been tested for homoskedasticity using the Breusch Pagan test, for Gaussian residuals using the Shapiro–Wilk test and linearity using a rainbow test. To account for possible multicollinearity between the patient characteristics, variance inflation factor values were calculated and to identify potential outliers or leverage values, the Cook’s distance has been calculated for each linear model. Statistical analyses were performed using R (version 4.2.3) [28]. A *p*-value < 0.05 was considered statistically significant.

To determine the BV, the widely employed Nadler formula was used, which uses sex, height in m (H), and body weight in kg (W) [29]:

$${Men}:\,{Blood\; Volume}=(0.3669\times {H}^{3})+(0.03219\times W)+0.6041$$

$${Women}:{Blood\; Volume}=(0.3561\times {H}^{3})+(0.03308\times W)+0.1833$$

## Results

### Patients baseline characteristics

**Fig. 1:**
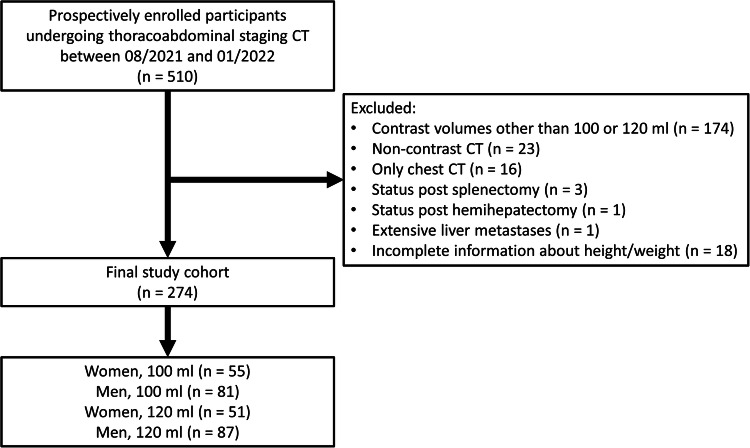
Inclusion and exclusion flow chart

A total of 510 consecutive patients were primarily enrolled. Thereof, 236 patients were excluded due to the following reasons: Contrast volumes other than 100 or 120 mL (*n* = 174), non-contrast CT (*n* = 23), only chest CT (*n* = 16), missing height or weight (*n* = 18), status post splenectomy (*n* = 3), hemihepatectomy (*n* = 1) and extensive liver metastases (*n* = 1), see Fig. 1. The final study cohort comprised 274 patients (168 males) with thoracoabdominal contrast-enhanced CT in portal-venous phase. Mean age was 68.0 ± 11.9 years, mean BMI was 26.0 ± 4.8 kg/m^2^. A total of 136 patients received 100 mL, and 138 patients received 120 mL intravenous CM. Patient characteristics and CT radiation dose parameters are summarized in Table 1.

**Table 1:** Study baseline characteristics including clinical and CT radiation dose parameters

Total *n* = 274			
**Clinical parameters**			
Age (years)	68.04 (± 11.87)		
Male	168 (61%)		
Body height (cm)	170.7 (± 8.91)		
Body weight (kg)	75.84 (± 15.41)		
BMI (kg/m²)	26 (± 4.78)		
**CT radiation dose parameters**	**100 mL (*n*** = **136)**	**120 mL (*n*** = **138)**	***p*-value**
Mean CTDI_vol_ (mGy)	8.51 (± 2.79)	8.41 (± 2.78)	0.76
DLP (mGy*cm)	487.19 (± 190.73)	504.37 (± 183.13)	0.45
SSDE (mGy)	10.2 (± 2.33)	10.02 (± 2.28)	0.53

Values are mean ± standard deviation. CT radiation dose parameters are tested for differences in mean between 100 mL and 120 mL using a *t*-test

*BMI* body mass index, *CT* computed tomography, *CTDI* computed tomography dose index, *DLP* dose length product, *SSDE* size-specific dose estimate

### Imaging results

**Fig. 2:**
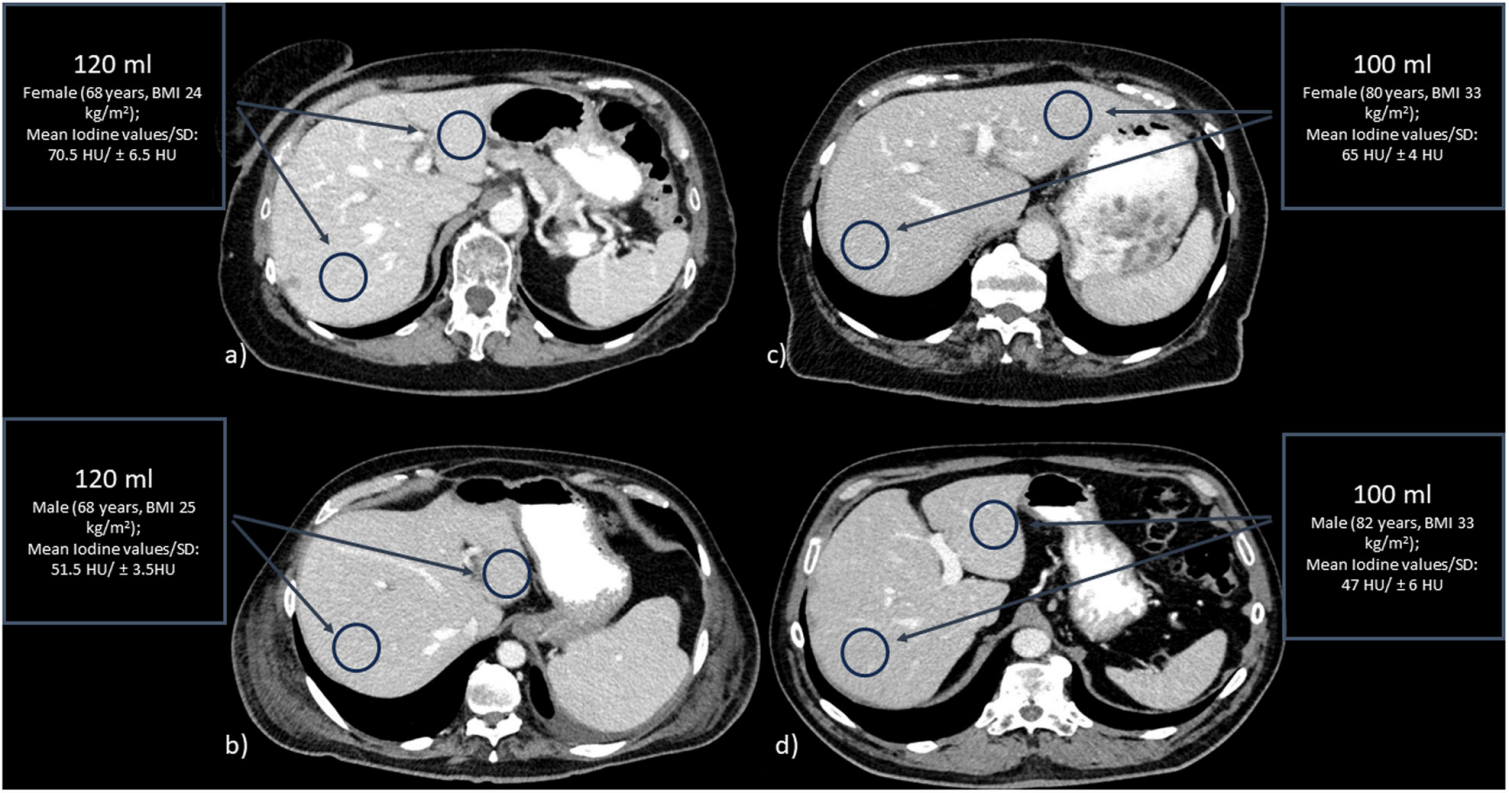
Sex-specific differences of iodine values in the liver. This figure shows the measured iodine values in the hepatic parenchyma using the example of a man and a woman with similar age and BMI for 120 mL (**a**, **b**) and 100 mL (**c**, **d**) of applied contrast medium. Notice the higher attenuation of the hepatic parenchyma and blood attenuation in females (upper row) compared to BMI- and age-matched males (lower row)

**Fig. 3:**
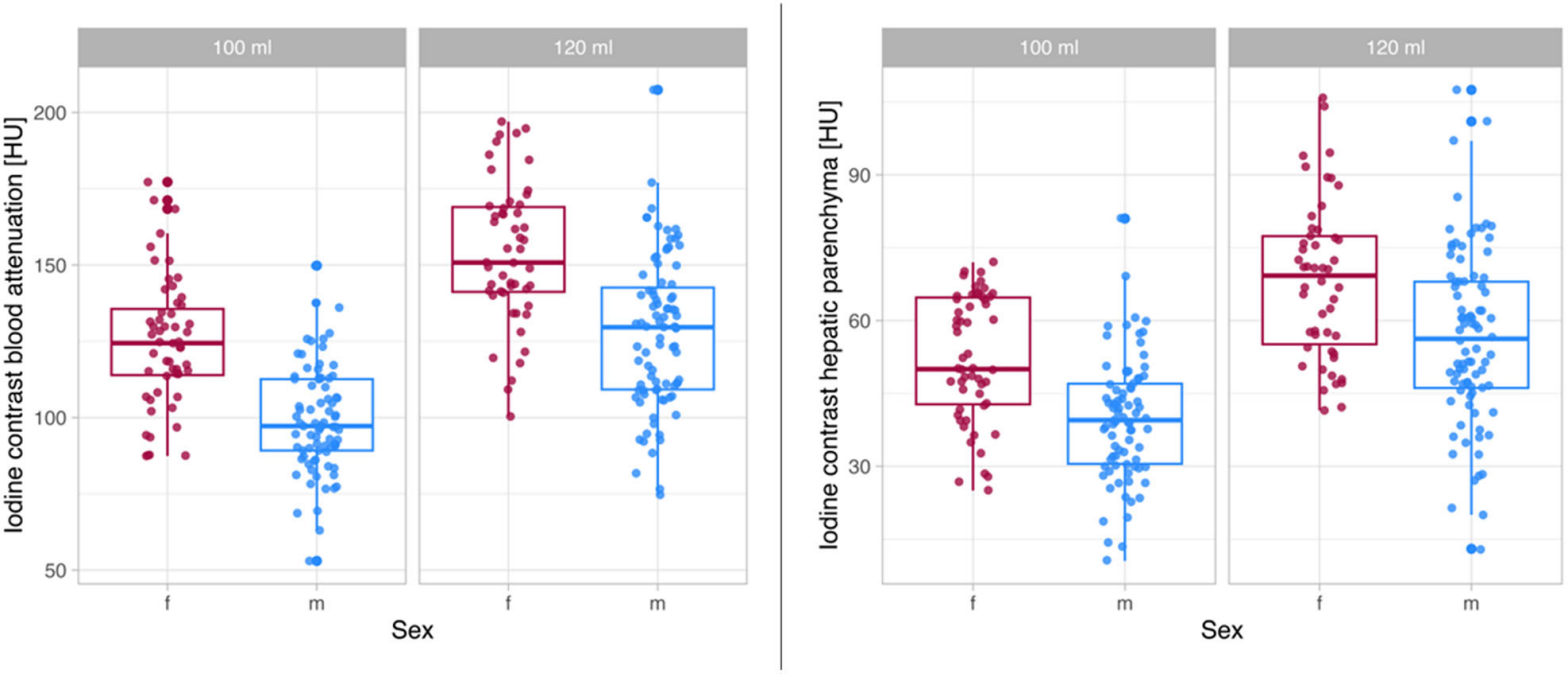
Iodine contrast values (HU) in blood attenuation and hepatic parenchyma separated by sex. The boxplots demonstrate the achieved iodine contrast (HU) in hepatic parenchyma and blood attenuation separated by sex, showing an observable difference between men and women

The iodine measurements performed for the different contrast groups (100 and 120 mL) demonstrate that the average iodine contrast was higher in women than in men in solid organs and in blood attenuation. Figure 2 shows the difference in the iodine contrast of a BMI and age-matched man and woman in a contrast-enhanced CT scan in portal-venous phase. In the 120 mL group, the iodine contrast of the hepatic parenchyma was, on average, 68 ± 16 HU in females and 57 ± 18 HU in men. In the 100 mL group, the average iodine contrast of the hepatic parenchyma was 52 ± 13 HU in females and 40 ± 13 HU in men. The results of the different achieved iodine contrast in blood attenuation and hepatic parenchyma, for both the 100- and 120-mL groups are shown in Fig. 3 (supplementary figures for spleen and portal vein, Supplementary Fig. 3.1). These boxplots visualize the difference between the HU in women and men by applying the same amount of CM. Table 2 provides detailed data on attenuation for different anatomical regions, sex and contrast volume.

**Table 2:** Sex-dependent achieved HU in different organs with 100 and 120 mL applied contrast media

	100 mL			120 mL		
Characteristics	Female (*n* = 55)	Male (*n* = 81)	*p*-value	Female (*n* = 51)	Male (*n* = 87)	*p*-value
Hepatic parenchyma HU mean	116 (± 14)	103 (± 15)	< 0.001^a^	125 (± 17)	116 (± 21)	0.014^a^
Hepatic parenchyma HU iodine	52 (± 13)	40 (± 13)	< 0.001^a^	68 (± 16)	57 (± 18)	< 0.001^a^
Hepatic parenchyma HU VNC	63 (± 7)	64 (± 8)	0.84^a^	56 (± 8)	59 (± 9)	0.048^a^
Spleen HU mean	125 (± 14)	108 (± 14)	< 0.001^a^	155 (± 21)	137 (± 23)	< 0.001^a^
Spleen HU iodine	72 (± 16)	52 (± 15)	< 0.001^a^	108 (± 21)	88 (± 25)	< 0.001^a^
Spleen HU VNC	53.2 (± 3.7)	56 (± 4.5)	< 0.001^a^	46.9 (± 5.1)	48.5 (± 4.8)	0.07^a^
Portal vein HU mean	190 (± 29)	164 (± 26)	< 0.001^a^	230 (± 37)	198 (± 32)	< 0.001^a^
Portal vein HU iodine	154 (± 29)	129 (± 129)	< 0.001^a^	188 (± 32)	159 (± 30)	< 0.001^a^
Portal vein HU VNC	36 (± 7)	35 (± 6)	0.29^a^	41 (± 10)	39 (± 8)	0.16^a^
Blood attenuation HU mean	165 (± 21)	141 (± 17)	< 0.001^b^	194 (± 24)	170 (± 23)	< 0.001^a^
Blood attenuation HU iodine	124 (± 21)	100 (± 18)	< 0.001^a^	154 (± 23)	128 (± 25)	< 0.001^a^
Blood attenuation HU VNC	40.1 (± 4.6)	41.7 (± 4.9)	0.07^a^	41 (± 7)	42 (± 6)	0.20^a^

*VNC* virtual non-contrast, *HU* Hounsfield Units, mean (± SD)

^a^ The characteristics are tested for differences in means using *t*-test

^b^ The characteristics are tested for differences in means using Welch test

### Variables influencing the iodine contrast

**Fig. 4:**
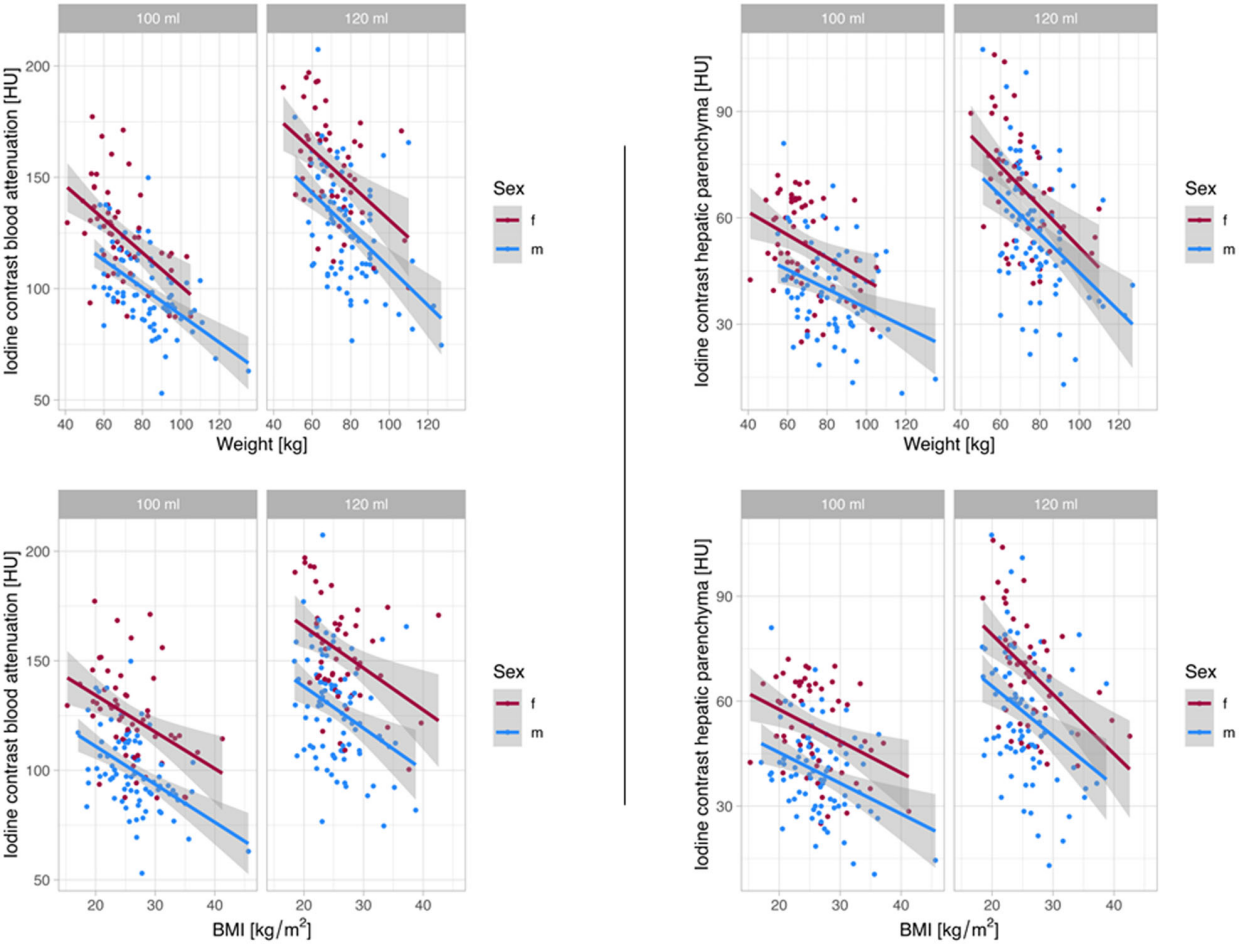
Sex-dependent iodine contrast values (HU) in the blood and the hepatic parenchyma corrected for body weight or BMI illustrated with linear regression for each sex corrected for body weight or BMI

Figure 4 illustrates that an identically applied volume of CM in patients with the same BMI or body weight leads to a sex-different enhancement in organs and the blood (supplementary figures for spleen and portal vein, Supplementary Fig. 4.1). With identical applied volume of CM, women, on average, achieve higher HU at the same BMI or body weight than men.

In the following, we investigated which variable (sex, age, BMI, height, body weight, volume of CM) has an influence on the iodine contrast obtained in the organs and the blood. In univariable analyses, all assessed covariables showed a significant influence on the achieved HU in the respective measured regions (see Table 3 for blood attenuation, supplementary tables for liver, spleen and portal vein, Supplementary Tables 3.1–3.3). In the regression, female sex was associated with 25 HU (95% CI: 20.19–30.76) more in blood attenuation than male sex with an identically applied CM volume. An increase in age by 1 year corresponded to an increase of 0.34 HU (95% CI: 0.09–0.59). With increasing BMI, there is a decrease in the achieved iodine contrast values. If the variables are examined in a multivariable model, the contrast values achieved relativize and the influence on the contrast enhancement decreases. While sex and body weight still have a significant influence on HU and thus remain independent variables, age and height lose their significance. Adding interaction terms of sex and age, height or weight did not improve the models in terms of the Akaike information criterion (AIC). To not reduce the power of the other variables in the model, the interaction terms were not considered further.

**Table 3:** Influence of different variables on the achieved iodine contrast in the blood attenuation

	Univariable model		Multivariable model	
	Estimate (95% CI)	*p*-value	Estimate (95% CI)	*p*-value
Age (years)	0.34 (0.09–0.59)	0.008	0.16 (−0.04 to 0.35)	0.11
BMI (kg/m^2^)	−1.78 (−2.37 to −1.19)	< 0.001	–	–
Weight (kg)	−0.91 (−1.07 to −0.75)	< 0.001	−0.67 (−0.83 to −0.50)	< 0.001
Height (cm)	−1.38 (−1.67 to −1.08)	< 0.001	−0.32 (−0.67 to 0.02)	0.07
Sex (m, baseline: f)	−25.43 (−30.76 to −20.19)	< 0.001	−15.05 (−20.95 to −9.15)	< 0.001
Blood volume (L)	−20.74 (−23.59 to −17.89)	< 0.001	–	–
CM volume (mL) (120 mL, baseline: 100 mL)	27.79 (21.83–33.76)	< 0.001	27.57 (23.06–32.08)	< 0.001

### Blood volume and the impact on iodine attenuation

**Fig. 5:**
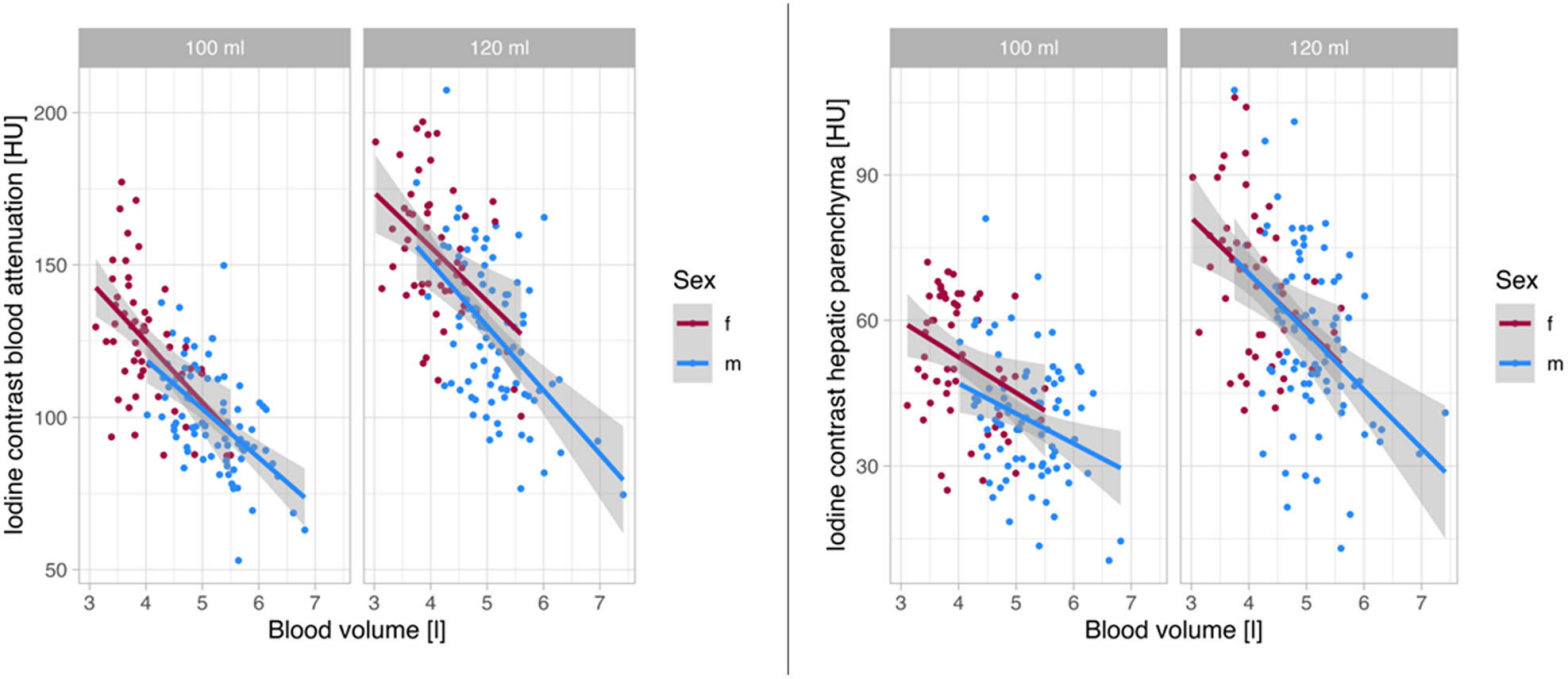
Sex-dependent iodine contrast values (HU) in the blood and hepatic parenchyma corrected for blood volume illustrated with linear regression for each sex corrected for blood volume

In the following, we examine the achieved HU in relation to the BV, calculated by Nadler. Figure 5 indicates that patients with an identically applied amount of CM and with the same BV have a similar contrast enhancement both in blood and hepatic parenchyma regardless of sex (refer to Supplementary Fig. 5.1 for spleen and portal vein).

Sex significantly influences the HU obtained in blood, hepatic parenchyma, spleen, and portal vein when correcting either for body weight, height, or BMI. In the multivariable model with BV, sex and applied amount of CM, sex no longer has a statistically significant influence on the iodine contrast of blood, hepatic parenchyma and portal vein. However, the iodine contrast obtained in the spleen remains significantly influenced by sex when correcting for BV (Table 4 for blood attenuation, Supplementary Tables 4.1–4.3 for hepatic parenchyma, spleen and portal vein).

**Table 4:** Linear model for the iodine contrast in the blood

Linear model with sex, CM and	*p*-value (sex m, baseline: f)
Blood volume (L)	0.09
Body weight (kg)	< 0.001
Body height (cm)	< 0.001
BMI (kg/m²)	< 0.001

## Discussion

In this study, we investigated whether sex has a significant influence on iodine contrast of the blood pool and organs in thoracoabdominal staging CT examinations using identical administration protocols for women and men. The main findings are: (1) When receiving identical volumes of CM, women show significantly higher attenuation values in the vessels and organs compared to men; (2) female sex is significantly associated with increased iodine contrast, independent of weight, height, age and contrast volume; (3) patient BV calculated using the Nadler method, which includes sex, height, and weight, has the potential to correct for sex-specific differences in iodine contrast.

The amount of intravenous CM applied in thoracoabdominal staging CT scans remains a controversial topic, as no general recommendations exist; thereof, a fixed dose of CM continues to be widely used [23, 25]. To our knowledge this is the first study demonstrating that iodine contrast in organs and the blood pool is significantly different in men and women when applying the same volume of CM in portal-venous staging CT. Our findings challenge the mainly used standardized or weight-adapted CM application. The results of our investigation underscore that women get a higher intravenous CM dose than necessary and are, in consequence, exposed to an increased risk in comparison to men. There are several possible explanations for our findings, such as the fact that the adipose tissue contributes little to dilution of the CM because of its small vascular or interstitial spaces, and therefore, the main amount of CM remains in the vessels and organs [7]. Additionally, women generally have higher amounts of body fat and less muscle mass than men [30]. Since women are at higher risk to suffer of severe side effects and some of these effects depend on the volume of CM, the application of unnecessary CM is an issue of absolute concern [12, 13, 15–17]. Further, a reduction of CM may lead to a decrease in overall healthcare costs [23, 24] and positively mitigate additional effects, such as the amount of excreted CM detected in drinking water. So far, there are no known serious consequences for the population or environment, but this shows that the existing wastewater treatment plants are not able to completely eliminate the excreted CM, which could be a growing problem due to the increasing number of CT examinations [31].

We suggest that the amount of CM should be sex-adapted in delayed CT scans. In this study, the iodine contrast in organs and the blood pool was not significantly influenced by sex when correcting for BV. Only the iodine contrast of the spleen sex remained significant after correction for BV, which may be due to its often inhomogeneous enhancement and the resulting difficulty in obtaining valid measurements [32]. The Nadler formula used in this study to calculate the BV is widely used in clinical practice, especially in intensive care medicine [29, 33, 34]. The influence of BV on the amount of CM applied has been controversial [6, 35].

Various studies already questioned the standardized application of CM in patients but with different results [6–8]. Kidoh et al favored the BSA to adjust the dose of CM in comparison to BV and lean body weight [6]. However, comparing this study to others is challenging due to the variety of formulas used to calculate BSA [36]. Additionally, this study did not investigate whether gender differences in contrast enhancement exist [6]. Kondo et al, on the other hand, advocated the use of lean body weight [7]. However, the studies of Svensson et al and Davenport et al argue for a weight-adjusted amount of CM [23, 37]. Svensson et al also revealed a sex difference in CM enhancement, but after adjustment for differences in body weight, height and age, the difference was no longer statistically significant [37]. The reason might be the small number of patients included in this study (*n* = 100) [37]. Thus, the optimal dose for applied intravenous CM remains an unresolved issue.

This study has several limitations. First, this is a retrospective single-center analysis of a prospectively acquired cohort. A more balanced cohort in terms of sex could further improve the results. Second, we only included patients who received 100- or 120-mL CM to achieve large subgroups to compare. Third, the PCD-CT technology with inherent spectral sensitivity allows the assessment of iodine contrast in each scan and made it possible to perform this study. An extension of this study to other (especially single-source) CT systems without material differentiation or true non-contrast scans as reference to calculate iodine contrast is complicated by this fact. Fourth, some studies have investigated the influence of cardiac function and blood pressure on contrast enhancement achieved in CT imaging, particularly in the arterial vessels, but less so in the venous phase or during hepatic enhancement [38–42]. Recently, studies have highlighted the differences in blood pressure between men and women, further emphasizing the importance of gender-specific medicine [43–45]. Fifth, potential other patient factors, such as kidney function, which might have an impact on the achieved contrast enhancement in portal venous phase, were not assessed in this study. Sixth, this study did not include a control group to compare the effects of standardized or individualized contrast agents. Further prospective studies with balanced sex cohorts are needed to confirm the results of this study.

In conclusion, this study provides evidence that standardized, or weight-adjusted dosing of iodinated CM in venous-phase thoracoabdominal CT scans may result in significantly higher contrast in women compared to men, exposing them to higher risks and resulting in higher costs. Applying CM based on a sex-specific BV formula is a promising approach to account for sex differences in iodine contrast. In addition, personalized CM administration may reduce healthcare costs, but more importantly, it may reduce the patient’s exposure to intravenously applied CM and its associated risks. A prospective study is set up to confirm the above results and to validate the approach.

## Supplementary information

ELECTRONIC SUPPLEMENTARY MATERIAL

## Data Availability

The datasets used and analyzed during the current study are available from the corresponding author upon reasonable request.

## Abbreviations

BMI: Body mass index
BSA: Body surface area
BV: Blood volume
CM: Contrast medium
CT: Computed tomography
PCD-CT: Photon-counting detector CT
VNC: Virtual non-contrast
