# Supplementary material for: Are we systematically overdosing women? Revisiting standardized contrast protocols for thoracoabdominal CT scans
Source: Eur Radiol. 2025 Jan 9;35(7):3729–38. doi: 10.1007/s00330-024-11329-8 (PMC12165885; doi:10.1007/s00330-024-11329-8)
Supplement: Supplementary file 1 — ELECTRONIC SUPPLEMENTARY MATERIAL [file 330_2024_11329_MOESM1_ESM.pdf]

Are we systematically overdosing women? Revisiting standardized contrast protocols for thoracoabdominal CT scans

ELECTRONIC SUPPLEMENTARY MATERIAL

Suppl. Figure 3.1 The boxplots demonstrate the achieved iodine contrast (HU) in spleen and portal vein separated by sex, showing an observable difference between men and women.

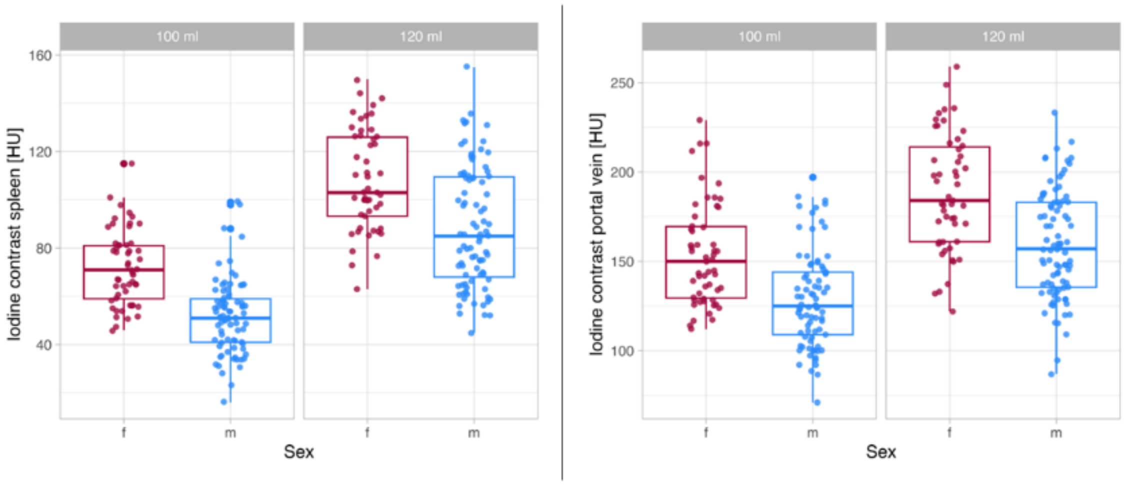

Suppl. Figure 4.1 demonstrates the achieved iodine contrast values (HU) in the spleen and portal vein with linear regression lines for each sex corrected for body weight and BM.

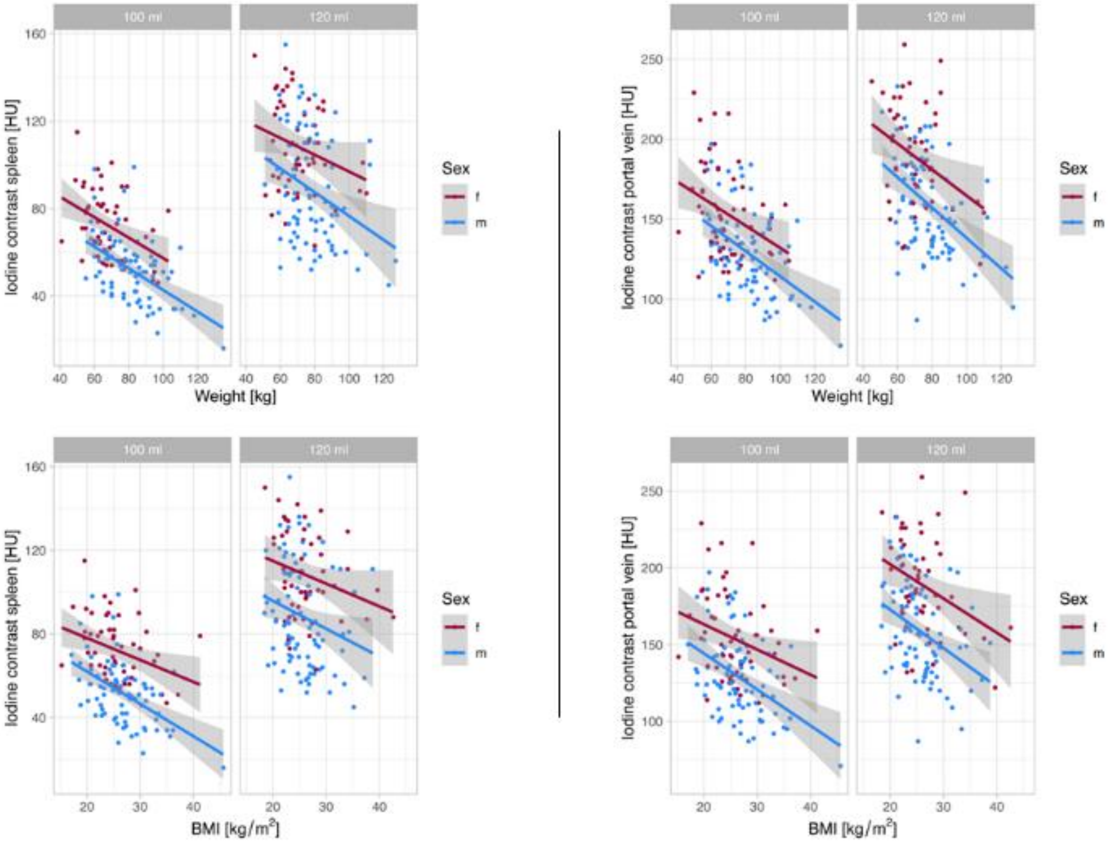

Suppl. Figure 5.1 demonstrates the achieved iodine contrast values (HU) in the spleen and portal vein with linear regression lines for each sex corrected for blood volume.

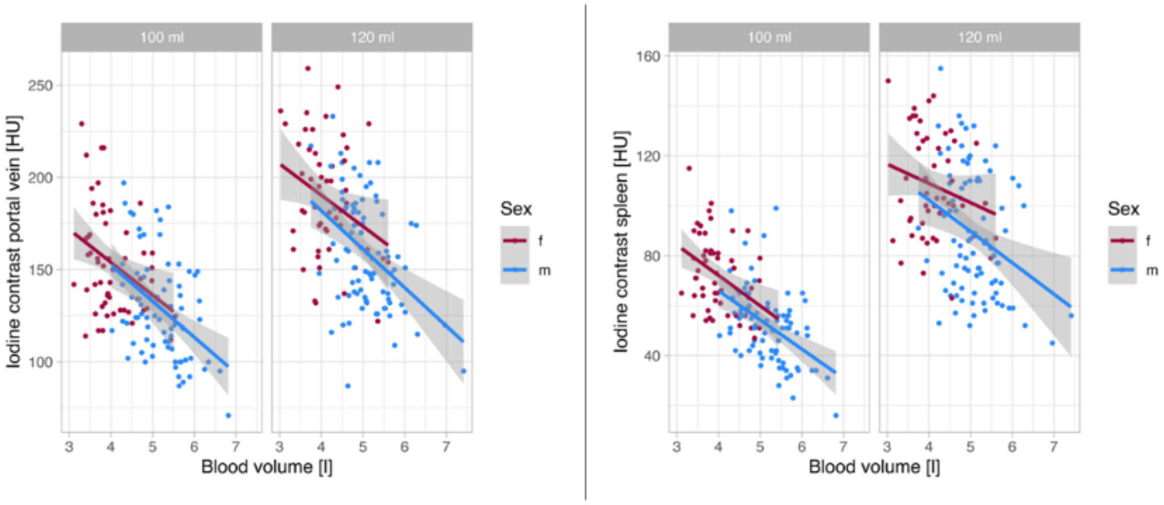

Suppl. Tables:

**Table 3.1** Influence of different variables on the achieved iodine contrast in the hepatic parenchyma				
	**Univariable model**		**Multivariable model**	
	**Estimate (95% CI)**	**p-value**	**Estimate (95% CI)**	**p-value**
Age [years]	0.14 (-0.03 – 0.30)	0.10	0.07 (-0.08 – 0.21)	0.35
BMI [kg/m^2]	-1.17 (-1.55 – -0.79)	< 0.001	-	-
Weight [kg]	-0.49 (-0.60 – -0.38)	< 0.001	-0.42 (-0.54 – -0.30)	< 0.001
Height [cm]	-0.56 (-0.76 – -0.35)	< 0.001	0.08 (-0.18 – 0.34)	0.54
Sex (m, baseline: f)	-12.21 (-15.92 – -8.50)	< 0.001	-9.03 (-13.41 – -4.64)	< 0.001
Blood volume [l]	-10.19 (-12.32 – -8.06)	< 0.001	-	-
CM volume [ml] (120 ml, baseline: 100 ml)	16.13 (12.25 – 20.00)	< 0.001	15.85 (12.50 – 19.19)	< 0.001

**Table 3.2** Influence of different variables on the achieved iodine contrast in the spleen				
	**Univariable model**		**Multivariable model**	
	**Estimate (95% CI)**	**p-value**	**Estimate (95% CI)**	**p-value**
Age [years]	0.31 (0.09 – 0.53)	0.006	0.21 (0.02 – 0.40)	0.031
BMI [kg/m^2]	-1.29 (-1.83 – -0.76)	< 0.001	-	-
Weight [kg]	-0.64 (-0.79 – -0.48)	< 0.001	-0.47 (-0.63 – -0.31)	< 0.001
Height [cm]	-0.88 (-1.16 – 0.60)	< 0.001	0.06 (-0.28 – 0.40)	0.74
Sex [m, baseline: f]	-19.93 (-24.83 – -15.03)	< 0.001	-15.49 (-21.37 – -9.61)	< 0.001
Blood volume [l]	-14.40 (-17.25 – -11.54)	< 0.001	-	-
CM volume [ml] (120 ml, baseline: 100 ml)	35.76 (30.48 – 41.04)	< 0.001	35.37 (30.91 – 39.82)	< 0.001

**Table 3.3** Influence of different variables on the achieved iodine contrast in the portal vein				
	**Univariable model**		**Multivariable model**	
	**Estimate (95% CI)**	**p-value**	**Estimate (95% CI)**	**p-value**
Age [years]	0.58 (0.26 – 0.89)	< 0.001	0.43 (0.16 – 0.71)	0.002
BMI [kg/m^2]	-2.17 (-2.93 – -1.41)	< 0.001	-	-
Weight [kg]	-1.00 (-1.21 – -0.78)	< 0.001	-0.78 (-1.01 – -0.55)	< 0.001
Height [cm]	-1.28 (-1.68 – -0.88)	< 0.001	0.10 (-0.38 – 0.59)	0.68
Sex (m, baseline: f)	-27.01 (-34.16 – -19.85)	< 0.001	-20.04 (-28.32 – -11.76)	< 0.001
Blood volume [l]	-21.59 (-25.67 – -17.51)	< 0.001	-	-
CM volume [ml] (120 ml, baseline: 100 ml)	31.02 (23.39 – 38.64)	< 0.001	29.94 (23.61 – 36.27)	< 0.001

**Table 4.1** Linear model for the iodine contrast in the hepatic parenchyma	
**Linear model with sex, CM and**	**p-value (sex m, baseline: f)**
Blood volume [l]	0.35
Body weight [kg]	<0.001
Body height [cm]	<0.001
BMI [kg/m^2]	<0.001

**Table 4.2** Linear model for the iodine contrast in the spleen	
**Linear model with sex, CM and**	**p-value (sex m, baseline: f)**
Blood volume [l]	0.019
Body weight [kg]	<0.001
Body height [cm]	<0.001
BMI [kg/m²]	<0.001

**Table 4.3** Linear model for the iodine contrast in the portal vein	
**Linear model with sex, CM and**	**p-value (sex m, baseline: f)**
Blood volume [l]	0.15
Body weight [kg]	<0.001
Body height [cm]	<0.001
BMI [kg/m²]	<0.001
